# Lung transplantation for suppurative diseases

**DOI:** 10.1186/1749-8090-8-S1-O238

**Published:** 2013-09-11

**Authors:** MN Samano, LM Fernandes, LG Abdalla, RHOB Teixeira, JE Afonso, SV Campos, DS Ferronatto, L Turaça, PM Pêgo Fernandes, FB Jatene

**Affiliations:** 1 Thoracic Surgery Department, Heart Institute (InCor), Hospital das Clínicas da Faculdade de Medicina da Universidade de São Paulo, São Paulo, Brazil; 2 Pneumology Department, Heart Institute (InCor), Hospital das Clínicas da Faculdade de Medicina da Universidade de São Paulo, São Paulo, Brazil; 3 Medical School, University of São Paulo, São Paulo, Brazil; 4 Thoracic and Cardiovascular Surgery Department, Heart Institute (InCor), Hospital das Clínicas da Faculdade de Medicina da Universidade de São Paulo, São Paulo, Brazil

## Background

Bronchiectasis may be associated to chronic respiratory failure, whereas lung transplantation (LTx) is the only long term treatment. According ISHLT registries, cystic fibrosis (CF) is the third cause for LTx (16.8%) and the best survival in five years (60%). Non-CF bronchiectasis corresponds to only 2.8% The aim of this study is to describe our experience with LTx for patients with suppurative diseases.

## Methods

Between 2000 and 2011, the charts of all patients who underwent LTx for suppurative diseases were reviewed.

## Results

Of 150 LTx performed, 59 patients (39.3%) had suppurative diseases, 29 (19.3%) were non-CF and 30 (20%) had CF. In non-CF group, mean age was 40.2±12.6 years, male predominance (58.3%) and all bilateral. Pulmonary hypertension was present in 50%, but cardiopulmonary bypass (CPB) used n 13.8%. None case of PGD3 was seen in this group. CF patients were younger (27.4+9.2 years), 53% males and almost all bilateral (97%). 30% had pulmonary hypertension, CPB used in 10% and 10% developed PGD3 . There were no difference in ventilation time, ICU and hospital stay. The survival rate at 1 and 5 years in CF was 92% and 77% and in non-CF was 84% and 75%. There was no difference according survival but these both groups showed better survival than other underlying diseases (p<0.001).

## Conclusions

Suppurative diseases are important source of patients for LTx in our program. The incidence of non-CF bronchiectasis is especially high corresponding to the forth indication. The survival rates of these both groups are excellent and comparable to other reports.

